# Multi-parametric flow cytometric and genetic investigation of the peripheral B cell compartment in human type 1 diabetes

**DOI:** 10.1111/cei.12362

**Published:** 2014-07-24

**Authors:** W S Thompson, M L Pekalski, H Z Simons, D J Smyth, X Castro-Dopico, H Guo, C Guy, D B Dunger, S Arif, M Peakman, C Wallace, L S Wicker, J A Todd, R C Ferreira

**Affiliations:** * JDRF/Wellcome Trust Diabetes and Inflammation Laboratory, Department of Medical Genetics, NIHR Cambridge Biomedical Research Centre, Cambridge Institute for Medical Research, University of CambridgeCambridge, UK; † Department of Paediatrics, School of Clinical Medicine, University of CambridgeCambridge, UK; ‡ Department of Immunobiology, King's College London, School of Medicine, Guy's HospitalLondon, UK

**Keywords:** B lymphocytes, human immunology, IL-2, IL-21, immunophenotyping, PTPN22, type 1 diabetes

## Abstract

The appearance of circulating islet-specific autoantibodies before disease diagnosis is a hallmark of human type 1 diabetes (T1D), and suggests a role for B cells in the pathogenesis of the disease. Alterations in the peripheral B cell compartment have been reported in T1D patients; however, to date, such studies have produced conflicting results and have been limited by sample size. In this study, we have performed a detailed characterization of the B cell compartment in T1D patients (*n* = 45) and healthy controls (*n* = 46), and assessed the secretion of the anti-inflammatory cytokine interleukin (IL)-10 in purified B cells from the same donors. Overall, we found no evidence for a profound alteration of the B cell compartment or in the production of IL-10 in peripheral blood of T1D patients. We also investigated age-related changes in peripheral B cell subsets and confirmed the sharp decrease with age of transitional CD19^+^CD27^−^CD24^hi^CD38^hi^ B cells, a subset that has recently been ascribed a putative regulatory function. Genetic analysis of the B cell compartment revealed evidence for association of the *IL2–IL21* T1D locus with IL-10 production by both memory B cells (*P* = 6·4 × 10^−4^) and islet-specific CD4^+^ T cells (*P* = 2·9 × 10^−3^). In contrast to previous reports, we found no evidence for an alteration of the B cell compartment in healthy individuals homozygous for the non-synonymous *PTPN22* Trp^620^ T1D risk allele (rs2476601; Arg^620^Trp). The *IL2–IL21* association we have identified, if confirmed, suggests a novel role for B cells in T1D pathogenesis through the production of IL-10, and reinforces the importance of IL-10 production by autoreactive CD4^+^ T cells.

## Introduction

Type 1 diabetes (T1D) is characterized by the autoimmune destruction of the pancreatic β cells, primarily by CD4^+^ and CD8^+^ T lymphocytes, resulting in dependency upon exogenous insulin [1,2]. In addition to T lymphocytes, macrophages have also been implicated in β cell death during the early stages of insulitis, while B cells have been shown to be one of the most abundant cell types to infiltrate the inflamed islets during late-stage insulitis [3,4]. Environmental factors, together with multiple polymorphic alleles of varying disease risk, determine susceptibility and progression of the disease [5]. The major histocompatibility complex (MHC), namely its human leucocyte antigen (HLA) class I and class II loci, constitutes the strongest known genetic risk factor for T1D. Candidate gene and genome-wide association studies have resulted in the identification of more than 45 additional genomic loci associated with disease risk (http://www.T1DBase.org), and several confirmed susceptibility genes [6,7]. The identification of four candidate genes directly involved in B cell receptor (BCR) signalling and B cell differentiation (*PTPN22*, *BACH2*, *PTPN2*, *SH2B3*), and three immunoregulatory cytokine genes (*IL10*, *IL2/IL21*, *IL27*) involved in the differentiation and function of specific B cell subsets, implicates a dysregulation in the B cell signalling pathway in the pathogenesis T1D.

Although T1D is thought to be predominantly a T cell-mediated disease, there is increasing evidence for a role of B cells in the initiation and progression of the disease. In the non-obese diabetic (NOD) mouse model of autoimmune diabetes, progression to overt diabetes is reduced in B cell-deficient mice [8]. The disease phenotype can be rescued only if B cell-deficient NOD mice are reconstituted with both bone marrow and B cells, which also leads to the development of T cell responses to T1D-specific autoantigens [9]. Furthermore, B cell depletion was also found to abrogate insulitis and insulin-specific autoimmune responses in NOD mice [10–12]. In humans, development of multiple disease-specific anti-islet autoantibodies remains the strongest known risk factor for the progression to clinical T1D [13], although it is generally believed that the autoantibodies themselves are not directly killing β cells through complement-mediated lysis. The latter assumption is supported by a lack of genetic overlap between genes and alleles that predispose to T1D and those that influence autoantibody positivity [14,15]. However, while it is clear that loss of B cell tolerance occurs, little is known regarding defects in peripheral B cell tolerance that may favour the development of T1D. One possibility that is commonly discussed is that B cells act as potent antigen-presenting cells to autoreactive T cells via sequestration of autoantigens by surface-expressed immunoglobulins [9,16,17].

Recently, a subset of transitional B cells described by the CD19^+^CD27^−^CD24^hi^CD38^hi^ phenotype have been referred to as regulatory B cells, and were characterized by the production of the anti-inflammatory cytokine interleukin (IL)-10 and a dependence on IL-21 signalling [18–22]. This B cell subset has been suggested to have inhibitory functions in a broad range of autoimmune diseases, such as systemic lupus erythematosus, rheumatoid arthritis, Sjögren's syndrome and autoimmune thrombocytopenia [18,19,23]. In T1D, the frequency of CD19^+^CD10^+^CD27^−^CD24^hi^CD38^hi^ cells has been reported to be increased in T1D patients, as well as in non-diabetic carriers of the *PTPN22* Trp^620^ (rs2476601; Arg^620^Trp) non-synonymous risk allele [24]. *PTPN22* is one of the strongest non-HLA genetic risk factors for T1D, and the non-synonymous Trp^620^ allele has been shown previously to impair BCR signalling by altering Ca(2+) flux in response to B cell stimulation [25]. Moreover, the Trp^620^ allele has also been shown to impair peripheral and central B cell tolerance, resulting in the accumulation of autoreactive B cells and up-regulation of genes involved in B cell activation, such as *CD40*, *TRAF1* and *IRF5* [26]. An increased frequency of CD5^+^ B cells, another subset which has been ascribed regulatory potential through the production of IL-10 [27,28], has also been reported to be increased in T1D patients immediately after disease diagnosis [29].

In the present study, we employed a comprehensive flow cytometry approach, using 15 fluorochrome-conjugated surface markers, to characterize the B cell compartment in the peripheral blood of T1D patients and healthy individuals, and assessed the role of six T1D loci implicated in B cell function, including the *PTPN22* Trp^620^ non-synonymous allele, in the regulation of this immune compartment. Furthermore, to investigate whether we could discern a systemic immunoregulatory defect in these patients, we also assessed the production of IL-10 in purified CD19^+^ B cells following IL-21 stimulation, which revealed an association between polymorphisms of the T1D locus *IL2–IL21* and IL-10 production in memory B cells and, in a follow-up analysis, in autoreactive T cells.

## Materials and methods

### Subjects

Adult long-standing (LS) T1D patients (*n* = 20) and healthy controls (HC; *n* = 21) matched for age (within 5-year age-bands), sex and time of sample preparation were recruited from the Cambridge BioResource (CBR-http://www.cambridgebioresource.org.uk). Newly diagnosed (ND) T1D patients (*n* = 25) and unaffected siblings (UAS) of other T1D probands (*n* = 25), matched for age, sex and time of sample preparation, were collected from the JDRF Diabetes–Genes, Autoimmunity and Prevention (D-GAP) study (http://paediatrics.medschl.cam.ac.uk/research/clinical-trials/). ND patients were characterized as having been diagnosed with T1D less than 2 years ago (with one exception of 42 months) and UAS were islet autoantibody-negative, and were not related to any T1D patient included in this study. All donors were of white ethnicity and all healthy controls were individuals without autoimmune disease (self-reported).

For the analysis of B cell phenotypes stratified by *PTPN22* genotype, 48 (non-overlapping) additional adult healthy donors homozygous for the *PTPN22* Arg^620^/Arg^620^ (*n* = 24) and Trp^620^/Trp^620^ (*n* = 24) genotypes were recruited from the CBR. Baseline characteristics for all participating subjects are summarized in Table 1.

**Table 1:** Baseline characteristics of study participants

		Age (years)			Duration of disease (months)		Autoantibody positivity* *n* (%)	
					
Cohort	*n*	Median	Range	Male *n* (%)	Median	Range	Single	Multiple
T1D (D-GAP)†	25	13	9–34	16 (64·0)	12	2–42	25 (100%)	22 (88)
T1D (CBR)‡	20	32	22–42	8 (40·0)	198	6–376	–	–
T1D (combined)	45	20	9–42	24 (53·3)	22	2–376	–	–
Unaffected relatives (D-GAP)	25	14	10–31	13 (52·0)	n.a.	n.a.	0	0
Healthy controls (CBR)	21	27	18–37	7 (33·3)	n.a.	n.a.	–	–
Healthy controls (combined)	46	18	10–37	20 (43·4)	n.a.	n.a.	–	–

Baseline characteristics for the study participants stratified by the study cohorts.

* Type 1 diabetes (T1D)-specific autoantibodies: GAD, IA2 and ZnT8.

† Newly diagnosed T1D patients (duration of disease ≤ 3 years) enrolled into the Diabetes – Genes, Autoimmunity and Prevention (D-GAP) study.

‡ Long-standing adult T1D patients enrolled from the Cambridge BioResource (CBR); n.a. = not applicable; – = data not available.

In order to replicate an association of the *IL10* genotype found in B cells, islet antigen-specific IL-10 secretion in CD4^+^ T cells was measured in a total of 266 individuals, including 85 newly diagnosed T1D patients and 181 unaffected siblings recruited from D-GAP.

### Ethics

All samples and information were collected with written and signed informed consent. The D-GAP study was approved by the Royal Free Hospital and Medical School research ethics committee; REC (08/H0720/25). Adult long-standing T1D patients and healthy volunteers were enrolled into the CBR. The study was approved by the local Peterborough and Fenland research ethics committee (05/Q0106/20).

### PBMC sample preparation

Blood volumes taken from each donor ranged between 25 and 50 ml (median volumes of 35 and 32·5 ml for donors enrolled from CBR and D-GAP, respectively). Peripheral blood mononuclear cells (PBMCs) were isolated by Ficoll gradient centrifugation and cryopreserved in 10% heat-inactivated human AB serum in aliquots of 10 × 10^6^ or 5 × 10^6^ PBMCs per vial at a concentration of 10 × 10^6^ cells/ml, as described previously [30]. Importantly, T1D patients and healthy controls were recruited contemporaneously and samples were processed and stored by the same investigators to prevent spurious findings caused by differential sample preparation. Median PBMC yields were 42·2 × 10^6^ and 56·7 × 10^6^ for CBR and D-GAP donors, respectively.

### Surface immunostainings

Cryopreserved PBMCs (10 × 10^6^) were thawed in a 37°C waterbath and resuspended in X-Vivo (Lonza, Castleford, UK) + 1% heat-inactivated, filtered human AB serum (Sigma, Poole, UK). Cell viability following resuscitation was assessed in 40 independent PBMC samples using the Fixable Viability Dye eFluor 780 (eBioscience, San Diego, CA, USA) and was found to be consistently very high (median = 95·6%; min = 86·8%, max = 98·2%) for all samples collected as part of the cohorts analysed in this study.

B cell surface immunostaining was performed on 10^6^ PBMCs using two independent immunostaining panels (∼750 000 stained with surface panel 1 and the remaining ∼250 000 stained with surface panel 2; see Supporting information, Table S1). To reduce the effects of experimental variation and other potential covariates, PBMC samples were processed in batches of a minimum of 10 samples per day. Each T1D patient was paired with one healthy donor matched for age (within 5-year age-bands), sex and time of sample preparation. Four T1D patients could not be sex-matched to a matching control, but as we found no differences associated with sex they were included in the paired analyses. One additional adult healthy donor from the CBR was not matched to a T1D patient and therefore excluded from the paired association analyses. PBMCs were stained for 1 h at 4°C with immunostaining panels 1 or 2, respectively (see Supporting information, Table S1).

To assess the reproducibility of the assessed surface immunostaining phenotypes, we have repeated the measurement of each assessed immune phenotype (with the exception of CD19^+^CD5^+^ B cells) using an independent PBMC sample from 18 donors, within 1–2 months of the original measurement.

### Cell culture and *in-vitro* stimulation

CD19^+^ B cells were purified by magnetic-activated cell sorting using the human CD19 positive selection kit (Miltenyi Biotec, Bisley, UK), from 9 × 10^6^ PBMCs and yielded an average of 300 000 CD19^+^ cells per donor. Cells (100 000–150 000 per well) were rested for 30 min at 37°C in RPMI medium supplemented with 10% fetal bovine serum (FBS), 2 mM L-glutamine and 100 μg/ml Pen-Strep in a 96-well round-bottomed plate (Greiner bio-one, Stonehouse, UK), and then cultured for 3 days at 37°C with anti-CD40 (200 ng/ml; Biolegend, London, UK), cytosine–phosphate–guanosine (CpG) (20 nM; InvivoGen, Stockport, UK) and either IL-21 (1 ng/ml or 100 ng/ml; Gibco, Paisley, UK) or culture medium alone (unstimulated controls). After 3 days, samples were activated with 50 ng/ml phorbol myristate acetate (PMA; Sigma-Aldrich, Poole, UK) and 500 ng/ml ionomycin (Sigma-Aldrich), 0·67 μl/ml Monensin GolgiStop (BD Biosciences, Oxford, UK), 1 μM CpG, and 10 μg/ml LPS (Sigma-Aldrich) for the last 5 h of culture. The higher dose of IL-21 (100 ng/ml) was found to induce IL-10 production by the majority of CD19^+^ B cells (median = 67·0%) and was therefore not analysed further in this study.

### Intracellular immunostainings

After activation, cells were harvested and stained with Fixable Viability Dye eFluor 780 for 20 min at 4°C. Cells were then stained with fluorochrome-conjugated antibodies against surface receptors (see Supporting information, Table S1) for 1 h at 4°C. Fixation and permeabilization was performed using forkhead box protein 3 (FOXP3) Fix/Perm Buffer Set (BioLegend) and B cells were then stained with a phycoerythrin (PE)-conjugated anti-IL-10 or isotype control antibody (IC) for 1 h at 4°C. Median cell viability was estimated to be 58·6% (min = 27·5%, max = 82·8%) following cell culture and *in-vitro* activation.

All experiments were performed in an anonymized, blinded manner without prior knowledge of disease state or genotype.

### Flow cytometry

**Figure 1:**
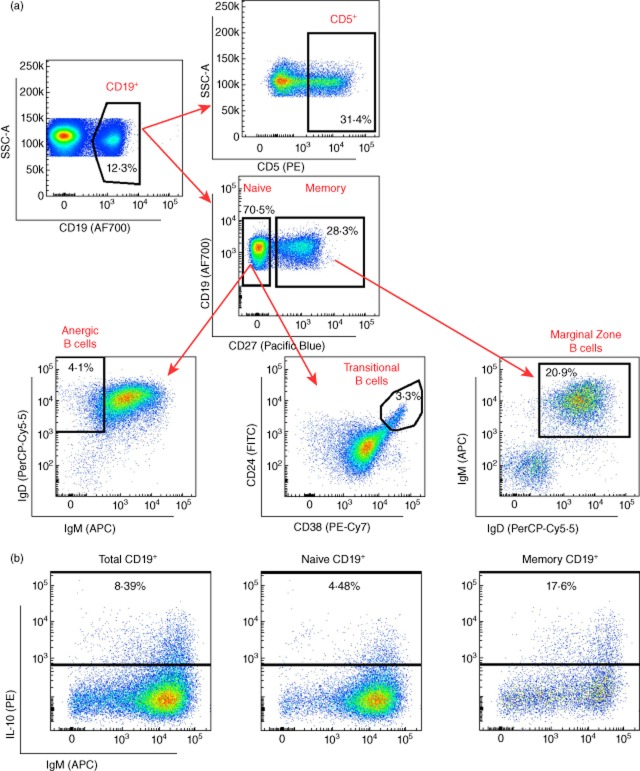
Delineation of peripheral B cell subsets. (a) Gating strategy for the delineation of the seven B cell subsets characterized in this study. Fluorochrome-conjugated surface markers used for the surface characterization of the subsets are shown in the respective plot. The initial CD19^+^ gate (CD19 *versus* side-scatter) was derived from a lymphocyte gate (defined on forward- and side-scatter) followed by single-cell discrimination. (b) Gating strategy for the characterization of the frequency of interleukin (IL)-10^+^ cells in total, naive and memory CD19^+^ populations using an intracellular immunostaining approach. Plots and frequencies shown in these plots correspond to the delineation of the subsets in one illustrative individual.

Immunostained samples were analysed using a BD Fortessa (BD Biosciences) flow cytometer with fluorescence activated cell sorter (FACS)Diva software (BD Biosciences). Flow cytometry data were exported in format 3·0 and analysed using FlowJo (Tree Star, Inc., Ashland, OR, USA). Compensation controls were generated using CompBeads (BD Biosciences) compensation beads. Cyto-Cal calibration beads (Thermo Scientific, Runcorn, UK) were used to assess instrument stability. Gating strategies, following doublet-cell exclusion for all assessed immune-cell populations, are depicted in Fig. 1. Dead-cell exclusion based on the Fixable Viability Dye was performed for the intracellular immunostainings.

### **Detection of β cell-specific IL-10^+^ CD4^+^ T** cells

Detection of IL-10 production by CD4^+^ T cells in response to peptide stimulation was carried out using an enzyme-linked immunospot (ELISPOT) assay, as described previously [31,32]. Peptides based on sequences of naturally processed and presented IA-2 (709-736, 752-775 and 853-872), proinsulin (C13-32, C19-A3 and C22-A5) and glutamic acid decarboxylase (GAD)-65 epitopes (335-352 and 555-567), as well as overlapping regions of the insulin B (1-20; 6-25 and 11-30) and A chain (1-21), were synthesized by fluorenylmethyloxycarbonyl chloride (Fmoc) chemistry and purified by high performance liquid chromatography (HPLC) (Thermohybaid, Ulm, Germany).

All assays were performed blinded to the clinical status of the donor. Data are expressed as the mean number of spots per triplicate and compared with the mean spot number in the presence of diluent alone (stimulation index: SI).

### Statistical analyses

Statistical analyses were performed using Prism software (GraphPad, San Diego, CA, USA) and Stata (http://www.stata.com). Association of each assessed immune phenotype with T1D was calculated using a two-tailed paired *t*-test. For IL-10 secretion phenotypes, which showed a strong right skew that violated the assumption of normality, the phenotypes were log-transformed before statistical testing. The effect of covariates, including age (continuous), sex (categorical), batch number (categorical) and duration of disease (continuous) on the assessed immune phenotypes was assessed using linear regression. Association of the B cell phenotypes with genotype at the six T1D loci implicated in B cell function was also performed by linear regression, assuming an additive effect model for the effect of the minor allele (coded as 0, 1 or 2 according to the number of minor alleles present) and including age, sex and batch as covariates. Interaction of age and disease status was tested by analysis of variance (anova) comparison of linear regression models allowing either equal or different effects of age in T1D patients and healthy controls.

Where multiple measurements of IL-10 secretion on CD4^+^ T cells after exposure to different peptides were available from the same individual, we dealt with the resulting intra-individual correlation within the same regression framework, but using a robust clustered variance estimator, with clusters defined by individuals.

For the analysis of the effect of *PTPN22* rs2476601 (Arg^620^Trp), eight samples (three Arg^620^/Arg^620^ and five Trp^620^/Trp^620^) showed compromised cell viability after thawing (>50% dead cells), and were excluded from the analysis. Exclusion of these eight samples compromised the paired structure of the original assay design, therefore association of each assessed phenotype with *PTPN22* Arg^620^Trp genotype was tested using linear regression models in the remaining 40 healthy donors (21 Arg^620^ homozygotes and 19 Trp^620^ homozygotes), including age and sex as covariates. Significance was assessed with a likelihood ratio test.

To estimate the power of our current study to detect increasing changes in the mean frequency of CD19^+^CD27^−^CD24^hi^CD38^hi^ cells between T1D patients and healthy controls, we assumed a reference mean frequency of 5·95% in controls, as observed in our study, and tested the power to detect a 1, 5, 10, 15, 20, 25, 30, 35, 40, 45 or 50% increase in T1D patients. The power calculation was based on a two-tailed *t*-test for a sample of 45 T1D patients and 46 healthy controls at 1 and 5% significance thresholds, and standard deviations of 2·83 and 3·62 in healthy controls and T1D patients, respectively, as observed in our study.

We estimated the power to detect a difference in CD19^+^CD27^−^CD24^hi^CD38^hi^ B cell frequency according to *PTPN22* genotype using the pwr.t2n.test function in the r [33] package pwr [34]. To estimate our power to replicate the published association, we assumed a difference per Trp^620^ allele of 1·04%, as reported by Habib *et al*. [24], and a common standard deviation of 2·05, which is the pooled estimate of standard deviation reported by Habib *et al*. [24]. To estimate the number of samples required to confirm the association if the effect is nearer our estimated effect, we assumed a per allele difference of 0·415% and a pooled standard deviation of 1·5.

## Results

### Delineation of peripheral B cell subsets

Seven major CD19^+^ B cell subsets were discriminated by flow cytometry in PBMCs from 91 donors, namely 45 T1D patients and 46 healthy controls (Fig. 1a and Supporting information, Table S2). All assessed B cell phenotypes were found to be highly reproducible in two independent measurements obtained from 18 different subjects (ρ > 0·77; Supporting information, Table S2). The only notable exception was the CD19^+^CD27^−^ immunoglobulin D (IgD)^+^IgM^−^ anergic B cell subset (ρ = 0·359; Supporting information, Table S2), which was therefore not assessed further in this study. To investigate a putative immunoregulatory defect in T1D patients, we assessed the frequency of two B cell subsets with suspected regulatory function, CD19^+^CD27^−^CD24^hi^CD38^hi^ transitional B cells and CD19^+^CD5^+^ (CD5^+^ B cells), as well as CD19^+^CD27^+^IgD^+^IgM^+^ (marginal zone) B cells. In addition to surface immunophenotyping, we assessed the intracellular production of IL-10 by total (CD19^+^), naive (CD19^+^CD27^−^) and memory (CD19^+^CD27^+^) B cells cultured in the presence of IL-21, following *in-vitro* stimulation (Fig. 1).

### The peripheral B cell compartment in T1D

**Figure 2:**
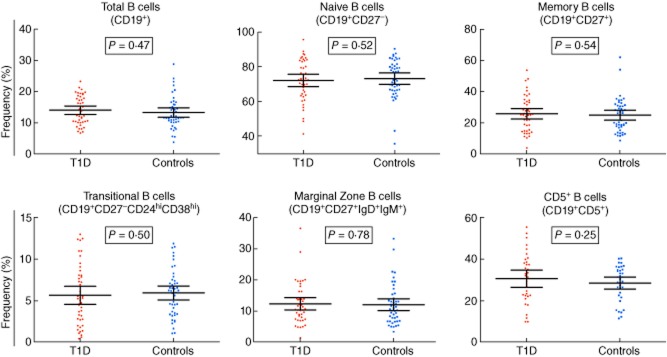
The peripheral B cell compartment is not altered in type 1 diabetes (T1D) patients. The frequency of the six B cell subsets robustly characterized in this study was compared between 45 T1D patients (long-standing and newly diagnosed; depicted by red circles) and 45 healthy donors matched for age, sex and time of sample preparation (depicted by blue squares). *P*-values were calculated using a two-tailed paired *t*-test. Horizontal bars represent the mean frequency in each genotype group and error bars depict the 95% confidence interval of the mean. Additional data from the statistical analysis are provided in Table 2.

**Figure 3:**
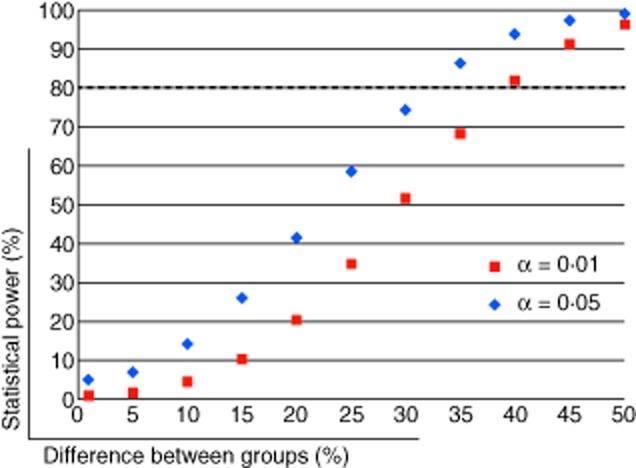
Statistical power calculation. Statistical power calculation was performed using the observed mean and standard deviation for the frequency of CD19^+^CD27^−^CD24^hi^CD38^hi^ B cells in the 46 healthy controls and 45 type 1 diabetes (T1D) patients assessed in this study. The plot depicts the statistical power (*y*-axis) of this study to detect an increasing difference in the mean frequency of this subset between the two groups (represented in the *x*-axis) using a two-tailed *t*-test at a 1% (α = 0·01; depicted by red squares) or 5% (α = 0·05; depicted by blue diamonds) significance level. The horizontal dotted line represents the 80% power threshold.

**Figure 4:**
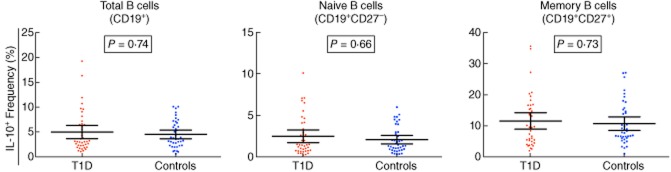
Interleukin (IL-10) production is not affected in B cells from type 1 diabetes (T1D) patients. Frequency of IL-10^+^ cells in total, naive and memory CD19^+^ B cells in T1D patients (*n* = 40; depicted by red circles) and healthy controls matched for age, sex and time of sample preparation (*n* = 40; depicted by blue squares). Frequency of IL-10 secreting cells was determined by intracellular immunostaining in purified CD19^+^ cells cultured for 3 days with IL-21 and following stimulation with cytosine–phosphate–guanosine (CpG), lipopolysaccharide (LPS), phorbol-12-myristate-13-acetate (PMA) and ionomycin. *P*-values were calculated using a two-tailed paired *t*-test. Data were log-transformed prior to statistical testing to correct for a strong right skew affecting the assumption of normality. Horizontal bars represent the mean frequency in each genotype group and error bars depict the 95% confidence interval of the mean. Additional data from the statistical analysis are provided in Table 2.

We first investigated the association of the delineated B cell phenotypes with T1D using a paired analysis of 45 T1D patients and 45 healthy donors, matched for age, sex and time of sample preparation. We found no evidence for a systematic difference in the frequency of any of the assessed B cell subsets in T1D patients (Fig. 2 and Table 2). In particular, we found no difference in the frequency of naive [*P* = 0·52, mean of differences = −1·15, 95% confidence interval (CI) = −4·71 to 2·41] or memory (*P* = 0·54, mean of differences = 1·04, 95% CI = −2·38 to 4·46) B cells or in the two subsets of B cells with a suspected regulatory function, CD19^+^CD27^−^CD24^hi^CD38^hi^ (*P* = 0·50, mean of differences = −0·36, 95% CI = −1·42 to 0·71) and CD5^+^ (*P* = 0·25, mean of differences = 2·34, 95% CI = −1·70 to 6·37) B cells, previously linked [24,29] with T1D (Fig. 2 and Table 2). Similarly, we also found no evidence for a variation in the frequency of these B cell subsets according to the time since T1D diagnosis (Supporting information, Table S3). Taking the observed distribution of CD19^+^CD27^−^CD24^hi^CD38^hi^ B cells in our study as a model, we have > 80% statistical power to detect alterations of more than 35 and 40% in the frequency of these cells between patients and healthy controls at a 5 and 1% significance level, respectively (Fig. 3). Because the distribution of the different B cell subsets was relatively uniform in our study (Fig. 4), the power calculations obtained for the CD19^+^CD27^−^CD24^hi^CD38^hi^ subset can be generalized more broadly for the other B cell subsets, and suggest that we have only adequate power to detect changes of > 35–40% in each of the assessed phenotypes.

Given the dependence of IL-21 signalling on the differentiation of CD19^+^CD27^−^CD24^hi^CD38^hi^ B cells, we also assessed the surface expression of IL-21R on these B cell subsets in T1D and healthy controls. However, we found no evidence of a differential expression of this receptor in T1D patients in any of the assessed B cell subsets (Table 2).

**Table 2:** Association analysis of B cell phenotypes with type 1 diabetes (T1D)

Phenotype	Pairs (*n*)	*P*-value	Mean of differences (95% CI)
B cell subsets			
Total CD19^+^ B cells	45	0·47	0·70 (−1·24, 2·65)
Naive B cells (CD27^−^)	45	0·52	−1·15 (−4·71, 2·41)
Memory B cells (CD27^+^)	45	0·54	1·04 (−2·38, 4·46)
Transitional B cells (CD27^−^CD24^hi^CD38^hi^)	45	0·50	−0·36 (−1·42, 0·71)
Marginal zone B cells (CD27^+^IgD^+^IgM^+^)	45	0·78	0·32 (−1·99, 2·62)
CD5^+^ B cells	35	0·25	2·34 (−1·70, 6·37)
IL-21R surface expression (MFI)			
Total CD19^+^ B cells	45	0·11	−96·7 (−217·0, 23·7)
Naive B cells (CD27^−^)	45	0·14	−73·78 (−173·0, 25·4)
Memory B cells (CD27^+^)	45	0·52	−7·4 (−30·4, 15·6)
Transitional B cells (CD27^−^CD24^hi^CD38^hi^)	45	0·24	89·6 (−61·9, 241·2)
Marginal zone B cells (CD27^+^IgD^+^IgM^+^)	45	0·58	−7·8 (−35·9, 20·3)
CD5^+^ B cells	n.a.	n.a.	n.a.
IL-10 production*			
IL-10^+^ total CD19^+^ B cells	40	0·74	0·018 (−0·087, 0·123)
IL-10^+^ naive B cells	40	0·65	0·025 (−0·086, 0·135)
IL-10^+^ memory B cells	40	0·73	0·020 (−0·096, 0·136)

*P*-values were calculated using a two-tailed paired *t*-test comparing the mean of the B cell phenotypes in T1D patients and healthy donors matched for age, sex and time of sample preparation.

* Statistical tests were performed on log-transformed data because interleukin (IL)-10 phenotypes showed a strong right skew. CI = 95% confidence interval; MFI = mean fluorescence intensity; n.a. = not applicable.

To investigate a potential immunoregulatory defect in the B cell compartment of T1D patients, we next assessed the frequency of IL-10^+^ B cells by intracellular immunostaining in magnetically purified CD19^+^ B cells isolated from the same donors, and cultured in the presence of IL-21 for 3 days to induce the differentiation of IL-10-secreting CD19^+^CD27^−^CD24^hi^CD38^hi^ B cells, as described previously [20]. We found no evidence for a functional difference in the production of IL-10 in CD19^+^, naive and memory B cells isolated from T1D patients compared to healthy controls (Fig. 4 and Table 2), or for an alteration in the production of this cytokine according to the duration of the disease (Supporting information, Table S3).

### Age-dependent changes in the composition of the human peripheral B cell compartment

**Figure 5:**
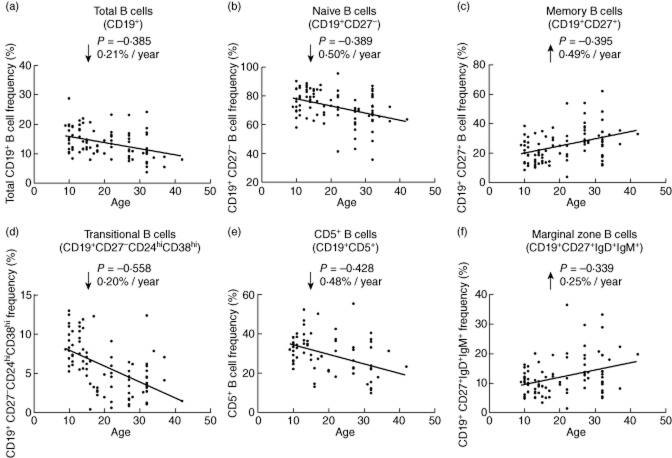
Association of B cell subsets with age. Scatter-plots depict the linear regression of the effect of age (represented on the *x*-axis) on the frequency of total CD19^+^ (a), naive CD19^+^ (b), memory CD19^+^ (c), transitional CD19^+^CD27^−^CD24^hi^CD38^hi^ (d), CD5^+^ (e) and CD19^+^CD27^+^IgD^+^IgM^+^ marginal zone (f) B cells (represented on the *y*-axis) in all assessed 91 donors. Additional data from the statistical analysis are provided in Table 3. ρ = correlation coefficient.

Age is known to have a strong effect on the differentiation of the adult immune system. Given the lack of association of T1D with the assessed B cell phenotypes, we next performed a linear regression analysis, combining all 91 donors included in this study, to identify age-related changes in the differentiation of the peripheral B cell compartment. The frequency of circulating total CD19^+^ B cells showed a strong negative correlation with age (ρ = −0·385, *P* = 1·63 × 10^−4^; Fig. 5a and Table 3). This variation corresponded to an average decrease of 0·21% in CD19^+^ B cells per year (Fig. 5a). We also confirmed the significant decrease of naive (ρ = −0·389, *P* = 1·40 × 10^−4^) and the concomitant increase of memory B cells (ρ = 0·395, *P* = 1·07 × 10^−4^) with age (Fig. 5b,c and Table 3). Consistently, we observed a negative correlation with age in the two naive B cell subsets that exhibit suspected regulatory properties – CD5^+^ B cells (ρ = −0·428, *P* = 2·21 × 10^−4^) and CD19^+^CD27^−^CD24^hi^CD38^hi^ B cells (ρ = −0·558, *P* = 9·09 × 10^−9^), while the memory subset of marginal zone B cells showed a positive correlation with age (ρ = 0·339, *P* = 1·01 × 10^−3^; Fig. 5d–f and Table 3). The negative correlation with age was particularly noticeable in CD19^+^CD27^−^CD24^hi^CD38^hi^ B cells, where age explained more than 31% of the trait's variance (*R*^2^ = 31·1%). This variation reflected a sharp reduction of the CD19^+^CD27^−^CD24^hi^CD38^hi^ B cell frequency from an average 8·7% in individuals aged less than 15 years to 3·7% in individuals aged more than 30 years, and corresponded to an average decrease of 0·2% per year (Fig. 5b and Table 3). The decreased frequency of the peripheral B cell subsets with age was even more apparent when analysing the absolute number of B cells in 55 of the 91 donors, where full blood counts were assessed (Supporting information, Fig. S1). These data provide a quantitative measure of the striking age-dependent changes observed in the absolute numbers of CD19^+^ cells, and demonstrate that there is a rapid decline in peripheral B cell numbers with age, which is particularly pronounced in naive (CD27^−^) subsets (Supporting information, Fig. S1).

**Table 3:** Age-dependent changes of peripheral B cell subsets

Phenotype	*n*	*P*-value	β (95% CI)
Total CD19^+^ B cells	91	1·63 × 10^−4^	−0·21 (−0·31, −0·10)
Naive B cells (CD27^−^)	91	1·40 × 10^−4^	−0·50 (−0·74, −0·25)
Memory B cells (CD27^+^)	91	1·07 × 10^−4^	0·49 (0·25, 0·73)
Transitional (CD27^−^CD24^hi^CD38^hi^)	91	9·09 × 10^−9^	−0·20 (−0·27, −0·14)
Marginal zone B cells (CD27^+^IgD^+^IgM^+^)	91	1·01 × 10^−3^	0·25 (0·10, 0·39)
CD5^+^ B cells	70	2·21 × 10^−4^	−0·48 (−0·73, −0·24)

Effect of age was calculated using a linear regression model. β = beta coefficient and 95% confidence interval (CI) of the linear regression model.

The rate of age-dependent changes did not differ between T1D patients and healthy controls (Supporting information, Fig. S2), indicating that the notable variation in the frequency of B cell subsets with age is not a consequence of a disease-specific mechanism, nor is the decline a major predisposing factor for T1D.

### Genetic regulation of the B cell compartment

Among the T1D genomic risk loci identified by genome-wide association studies, at least six harbour candidate genes that have been implicated directly in B cell differentiation and function (*PTPN22*, *BACH2*, *PTPN2*, *IL2–IL21*, *IL10* and *SH2B3*; http://www.T1DBase.org). To investigate the potential genetic regulation of the assessed B cell phenotypes, we tested the association of these traits with the genetic risk variants in these six loci, taking into account the strong age-dependent effects observed in this study. This genetic association analysis was limited by sample size, particularly for variants with low minor allele frequency. To account for multiple testing we have therefore used a conservative *P*-value threshold of 1·39 × 10^−3^, based on a Bonferroni correction for 36 independent tests, to report significant association with genotype.

We found no evidence for a major genetic association with the frequency of peripheral B cell subsets (Table 4). For IL-10 production by B cells, we obtained evidence for association of the minor allele at *IL2–IL21* (rs2069763 G > T), which confers increased risk for T1D, with decreased production of IL-10 in memory B cells (*P* = 6·4 × 10^−4^; Table 5). This association equated to a reduction of 57·8% in the mean frequency of IL-10^+^ memory B cells in the rare [TT] homozygotes (mean = 5·09%; *n* = 11) compared to [GG] homozygotes (mean = 12·06%; *n* = 26). To further investigate the role of genetic variation in the *IL2–IL21* locus in the regulation of IL-10 secretion, we next sought replication of these findings in an independent data set of CD4^+^ T cells stimulated with β cell-specific antigens. We found a consistent association between the rs2069763[T] allele and decreased numbers of IL-10^+^ islet antigen autoreactive CD4^+^ T cells (*P* = 2·9 × 10^−3^; Table 5), which supports a common regulatory mechanism for the production of this anti-inflammatory cytokine in both cell types mediated by the rs2069763 polymorphism in the *IL2–IL21* locus. Similarly to the genotype effect observed in the B cell data set, we found that the association in autoreactive CD4^+^ T cells corresponded to a 28·8% decrease in IL-10^+^ T cells in [TT] homozygotes (*n* = 1064) compared to [GG] homozygotes (*n* = 335).

**Table 4:** Genetic association of B cell-related T1D loci with the frequency of peripheral B cell subsets

					Naive B cells (CD27)		Memory B cells (CD27^+^)		Transitional B cells (CD27^−^CD24^hi^CD38^hi^)		Marginal zone B cells (CD27^+^IgD^+^IgM^+^)		CD5^+^ B cells	
									
Genetic locus	SNP	MAF*	OR†	*n*	*P*	β (95% CI)	*P*	β (95% CI)	*P*	β (95% CI)	*P*	β (95% CI)	*P*	β (95% CI)
*BACH2*	rs11755527	0·429	1·13	83	0·63	−0·75 (−4·11 to 2·61)	0·70	0·58 (−2·66 to 3·81)	0·46	0·34 (−0·64 to 1·32)	0·56	−0·53 (−2·54 to 1·47)	0·72	−0·58 (−4·05 to 2·88)
*PTPN2*	rs45450798	0·158	1·20	85	0·014	4·42 (0·61 to 8·24)	0·012	−4·34 (−8·00, −0·68)	0·42	−0·43 (−1·57 to 0·72)	3·6 × 10^−3^	−3·13 (−5·39, −0·88)	0·97	0·06 (−4·12 to 4·25)
*PTPN22*	rs2476601	0·117	2·05	86	0·99	0·02 (−4·68 to 4·72)	0·98	0·06 (−4·46 to 4·58)	0·52	0·41 (−0·97 to 1·78)	0·88	0·21 (−2·68 to 3·10)	0·23	−2·59 (−7·14 to 1·97)
*SH2B3*	rs3184504	0·445	1·28	82	0·16	2·07 (−1·06 to 5·20)	0·14	−2·07 (−5·09 to 0·96)	0·46	−0·32 (−1·24 to 0·61)	0·07	−1·49 (−3·28 to 0·30)	0·46	−1·15 (−4·55 to 2·24)
*IL10*	rs3024505	0·178	0·84	87	0·33	1·81 (−2·19 to 5·83)	0·34	−1·71 (−5·56 to 2·15)	0·45	−0·41 (−1·56 to 0·75)	0·27	−1·25 (−3·66 to 1·16)	0·23	2·27 (−1·73 to 6·27)
*IL2–IL21*	rs2069763	0·342	1·13	85	0·38	−1·27 (−4·40 to 1·86)	0·36	1·27 (−1·74 to 4·28)	0·66	0·18 (−0·71 to 1·08)	0·77	1·46 (−1·31 to 4·24)	0·74	0·51 (−2·85 to 3·87)

Association of the assessed B cell phenotypes with genotype at six B cell-related type 1 diabetes (T1D) loci was calculated using a linear regression model assuming an additive effect model for the effect of the minor allele and including age, sex and batch as covariates. A *P*-value threshold of 1·39 × 10, based on the Bonferroni correction for 36 independent tests, was set to report association with genotype (tests for the association with the frequency of CD27^−^ naive and CD27^+^ memory B cells were not counted twice, given their near-perfect negative correlation).

* Minor allele frequency (MAF) in the HapMap CEU population.

† Odds ratio (OR) of the minor allele for the association with T1D (http://www.T1DBase.org).

SNP = single nucleotide polymorphism; *n* = number of individuals; β = beta coefficient and 95% confidence interval (CI) of the linear regression model.

**Table 5:** Genetic regulation of interleukin (IL)-10 production

B cell subsets*									

						IL-10^+^ naïve B cells		IL-10^+^ memory B cells	
							
Genetic locus	SNP	MAF†	Minor allele	OR‡	*n*	*P*	β (95% CI)	*P*	β (95% CI)
*BACH2*	rs11755527	0·429	G	1·13	73	0·72	−0·03 (−0·24 to 0·17)	0·92	0·01 (−0·22 to 0·24)
*IL10*	rs3024505	0·178	A	0·84	77	0·95	0·01 (−0·24 to 0·25)	0·99	0·00 (−0·27 to 0·27)
*IL2–IL21*	rs2069763	0·342	T	1·13	75	0·09	−0·16 (−0·36 to 0·04)	6·4 × 10^−4^	−0·35 (−0·56, −0·14)
*PTPN2*	rs45450798	0·158	G	1·2	75	0·05	−0·22 (−0·45 to 0·02)	0·18	−0·16 (−0·42 to 0·10)
*PTPN22*	rs2476601	0·117	T	2·05	77	0·43	0·10 (−0·18 to 0·38)	0·25	0·17 (−0·14 to 0·47)
*SH2B3*	rs3184504	0·445	T	1·28	74	0·44	−0·07 (−0·28 to 0·13)	0·8	−0·03 (−0·25 to 0·19)

Antigen-specific CD4^+^ T cells§									

Genetic locus	SNP	MAF†	Minor allele	OR‡	nº samples	*P*	β (95% CI)		
*IL2–IL21*	rs2069763	0·342	T	1·13	2485	2·9 × 10^−3^	−0·04 (−0·07, −0·02)		

Association of the frequency of IL-10 B cells with genotype at six B cell-related type 1 diabetes (T1D) loci was calculated using a linear regression model assuming an additive effect model for the effect of the minor allele and including age, sex and batch as covariates.

* Statistical tests were performed on log-transformed data because IL-10 phenotypes showed a strong right skew.

† Minor allele frequency in the HapMap CEU population.

‡ Odds ratio (OR) of the minor allele for the association with T1D (http://www.T1DBase.org).

§ Analysis of the effect of *IL2–IL21* on the production of IL-10 in CD4^+^ T cells was obtained in an independent cohort of 266 individuals. Numbers of IL-10^+^ cells were assessed by enzyme-linked immunospot (ELISPOT) following stimulation of CD4^+^ T cells with 12 peptide epitopes from four islet-specific antigens (see Methods for detail). *P*-value was calculated on log-transformed data by combining all individual measurements and dealing with the resulting intra-individual correlation within the same regression framework, but using a robust clustered variance estimator, with clusters defined by individuals. SNP = single nucleotide polymorphism; MAF = minor allele frequency; β = beta coefficient and 95% confidence interval (CI) of the linear regression model; *n* = number of individuals.

In addition to the association with *IL2–IL21*, we also observed suggestive evidence for the association of *PTPN2*, a gene encoding a protein phosphatase involved in the negative regulation of the c-Jun N-terminal kinase–signal transducer and activator of transcription (JAK-STAT) signalling pathway, with reduced frequency of CD27^+^IgD^+^IgM^+^ marginal zone B cells (*P* = 3·6 × 10^−3^; Table 4), although it just failed to reach our significance threshold.

### Association of *PTPN22* Arg^620^Trp with peripheral B cell subsets

**Figure 6:**
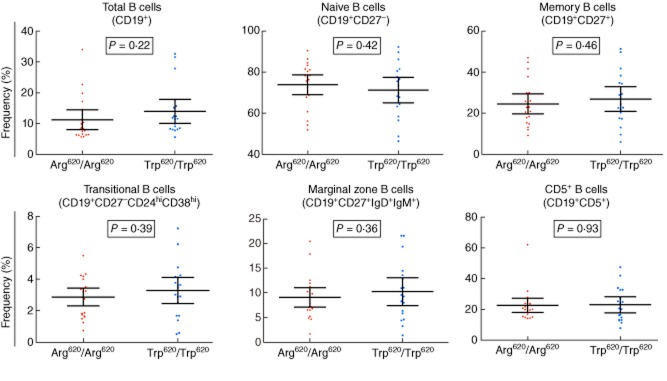
Frequency of peripheral B cell subsets is not affected by the *PTPN22* Arg^620^Trp non-synonymous polymorphism. Frequency of the six B cell subsets characterized in this study was compared between healthy donors homozygous for the Arg^620^ allele (*n* = 21; depicted by red circles) and age- and sex-matched healthy donors homozygous for the non-synonymous Trp^620^ allele (*n* = 19; depicted by blue squares). *P*-values were calculated using a linear regression analysis (see Materials and methods). Horizontal bars represent the mean frequency in each genotype group and error bars depict the 95% confidence interval of the mean. Additional data from the statistical analysis are provided in Table 2.

We were initially interested in the association of the T1D-associated *PTPN22* Arg^620^Trp (rs2476601) non-synonymous polymorphism because it had previously been associated with the frequency of transitional CD19^+^CD27^−^CD24^hi^CD38^hi^ B cells in heterozygous healthy donors [24]. To further investigate the effect of this T1D risk allele, we assessed the frequency of B cell subsets in an independent cohort of age-matched 21 Arg^620^ homozygous and 19 Trp^620^ homozygous healthy volunteers. We found no difference in the frequency of the assessed B cell phenotypes between the two genotype groups (*P* = 0·39; Fig. 6). This result was consistent with the lack of association of *PTPN22* with frequency of transitional CD19^+^CD27^−^CD24^hi^CD38^hi^ B cells both in the 86 immunophenotyped donors with available genotype data (*P* = 0·52; Table 4) or when restricting the analysis to the 46 non-diabetic donors (25 unaffected siblings and 21 adult healthy controls; *P* = 0·38). The very low frequency of Trp^620^ homozygous donors in the general population (< 1% in European and European-ancestry populations) has previously prevented the investigation of the frequency of CD19^+^CD27^−^CD24^hi^CD38^hi^ cells in Trp^620^ homozygotes. Importantly, by comparing the mean frequency of CD19^+^CD27^−^CD24^hi^CD38^hi^ B cells in the two homozygous groups, our data suggest that the estimated effect size corresponds to an absolute increase of 0·415% per copy of the Trp^620^ allele (95% CI = −0·540, 1·371), assuming an additive effect model, which suggests that any effect in Trp^620^ homozygous donors may be lower than reported previously [24].

## Discussion

In this study we have optimized a multi-parametric flow cytometric approach to characterize the B cell compartment in the peripheral blood of T1D patients and healthy individuals. To our knowledge, this is one of the largest studies to date attempting to characterize this immune compartment comprehensively in humans. We confirmed the previously reported negative correlation of total B cells with age [35,36]. In adults, the circulating B cell pool is maintained by output of cells from bone marrow and division of lymphocytes in the periphery, with the relative contribution of the two processes not well understood [37]. Thus, one likely cause of the decline in the frequency of total B lymphocytes with age is a decreased bone marrow output. Consistent with the observed reduction in the total CD19^+^ B cell frequency with age, we also observed a sharp decrease of two B cell subsets with naive properties: CD19^+^CD27^−^CD24^hi^CD38^hi^ transitional B cells and CD19^+^CD5^+^ B cells. These results are in agreement with previous studies [35,36,38] and confirm the strong age-dependence in the composition of these B cell subsets, which are much more abundant during childhood but decline rapidly thereafter. Interestingly, CD19^+^CD27^−^CD24^hi^CD38^hi^ and CD5^+^ B cells are two subsets with suspected regulatory properties via production of the anti-inflammatory cytokine IL-10 [18,19,27,39]. Such immune dysregulation resulting from the natural decline of these regulatory B cell subsets with age may, in fact, be a contributing factor to the increased propensity to infections, prevalence of autoimmune diseases and malignancy in older individuals [40,41].

In addition to age, we also found evidence for the association of the rs2069763[T] minor allele in the *IL2–IL21* locus, conferring increased risk for T1D, with reduced IL-10 production in memory B cells. Importantly, we replicated this effect in an independent data set of CD4^+^ T cells stimulated with β cell antigens. These data support a previously unrecognized functional role of the T1D-associated variant in this locus in the regulation of IL-10 production in both B and T cells. It is still unclear which is the causal susceptibility gene for T1D in this genomic locus, but both cytokines are known to be highly pleiotropic and play critical roles in the activation and regulation of the immune system. In addition to the six B cell-related genetic loci that we tested in this study, other T1D loci contain candidate genes that may either play a direct role in B cell function or regulate the function of another cell type that interacts with B cells. However, this study was not designed to perform a comprehensive investigation of all T1D-associated genetic loci. Further work will therefore be required to validate not only the association of *IL2–IL21* with IL-10 production, but also to assess the contribution of additional genetic variants to IL-10 production in different immune cell-types and under different IL-10-inducing activation conditions.

Despite this evidence for genetic regulation of the B cell compartment by a T1D risk locus, we found no evidence for a significant alteration of the assessed B cell subsets in T1D patients. Similarly, we did not find any evidence for the differential production of IL-10 by purified B cells from T1D patients following *in-vitro* stimulation with IL-21, which has been shown recently to drive the differentiation of IL-10-secreting effector CD19^+^CD27^−^CD24^hi^CD38^hi^ regulatory B cells in mice [20]. A limitation of this assay is that the production of IL-10 is dependent upon the stimulation and cell culture conditions. In this study, we have replicated experimental conditions shown previously to induce IL-10 secretion *in vitro*. However, we cannot exclude the possibility that T1D-specific alterations in IL-10-producing B cells could be manifested under different experimental conditions. Furthermore, we have assessed IL-10 production starting from an initial population of magnetically purified total CD19^+^ B cells, which is known to be a heterogeneous population. T1D-specific alterations in the IL-10 production potential could be restricted to only a specific B cell subset or to a minority of antigen-specific B cells in peripheral blood, which would not be identified in these conditions.

We also observed no differences in the frequency of CD19^+^CD27^−^CD24^hi^CD38^hi^ B cells between T1D patients and healthy controls, suggesting that there are no detectable B cell immunoregulatory alterations in peripheral blood of T1D patients after disease diagnosis. These results were in contrast with a previous study, reporting an increased frequency of CD19^+^CD10^+^CD27^−^CD24^hi^CD38^hi^ B cells in T1D patients [24]. This increased frequency mimicked the increase observed in the same study in the frequency of CD19^+^CD10^+^CD27^−^CD24^hi^CD38^hi^ B cells in healthy heterozygotes carriers of the *PTPN22* Trp^620^ allele compared to homozygous donors for the common Arg^620^ allele [24]. The B cell subset (CD19^+^CD27^−^CD24^hi^CD38^hi^) assessed in our study is comparable with the transitional subset (CD19^+^CD10^+^CD27^−^CD24^hi^CD38^hi^) analysed by Habib *et al*. [24]. While we did not use the CD10 marker, previous findings have reported that CD10 is homogeneously expressed at high levels in CD19^+^CD27^−^CD24^hi^CD38^hi^ cells [42]. One potential explanation for the lack of reproducibility of this finding in our population is a different age distribution of T1D patients and healthy controls (all adults) in Habib *et al*. [24] which, given the strong age-dependent effect in the frequency of CD19^+^CD27^−^CD24^hi^CD38^hi^ cells, could significantly affect the observed frequencies of this immune subset.

In our case–control study, sample selection was not based on *PTPN22* Arg^620^Trp genotype. However, including the *PTPN22* genotype as covariate in our linear regression analysis did not alter our results (data not shown). To further investigate the putative role of the non-synonymous Trp^620^ allele on the regulation of the CD19^+^CD27^−^CD24^hi^CD38^hi^ cells we were able to recruit the largest number (*n* = 19) of healthy homozygotes for the rare *PTPN22* Trp^620^ genotype to date and age- and sex-matched common Arg^620^ homozygotes from the Cambridge BioResource. Given the effect size for one copy of the Trp^620^ allele reported in Habib *et al*. [24], we would have expected to have more than 83% power to detect a differential frequency of this subset at a 5% significance level in our study, assuming an additive model for the effect each *PTPN22* Trp^620^ allele. We observed the same direction of association with increased CD19^+^CD27^−^CD24^hi^CD38^hi^ cells in Trp^620^ carriers, but an estimated per-allele effect of 0·415% (95% CI = −0·540 to 1·371) compared to 1·04% in Habib *et al*. [24]. These results are consistent with the established phenomenon of ‘winner's curse’, where estimated effect sizes tend to be inflated in samples where the effect is first detected. Thus, larger sample sizes will be required to confirm the putative association of *PTPN22* Trp^620^ with increased CD19^+^CD27^−^CD24^hi^CD38^hi^ B cell frequency: if the effect is nearer our per allele estimate, this would require 206 matched *PTPN22* Arg^620^/Arg^620^ – Arg^620^/Trp^620^ pairs at a 5% significance level.

CD19^+^CD5^+^ B cells have also been reported to be increased in T1D patients immediately after disease diagnosis (< 30 days after diagnosis) [29]. In our study, we found no evidence for the increased frequency of CD19^+^CD5^+^ B cells in either newly diagnosed or long-standing T1D patients compared to healthy controls. However, none of the T1D patients enrolled in our study were diagnosed within 30 days and, therefore, our findings are consistent with the observed lack of differences in CD5^+^ B cell frequency in T1D patients > 30 days since diagnosis reported by De Filippo *et al*. [29]. Further studies focusing specifically at the time of disease onset will be required to investigate a possible pathogenic role of CD19^+^CD5^+^ in the onset of disease.

Our data indicate that after disease diagnosis there are no significant changes affecting the delineation of B cell subsets in peripheral blood, thus illustrating the potential limitation of similar immunophenotyping studies in humans to robustly identify small phenotypical differences. To circumvent or minimize these pitfalls, it is critical to reduce all sources of technical variation to mitigate systematic batch effects and ensure the generation of high quality experimental data. In our study, given the observed intra-individual variation and sample size, we were adequately powered (80%) to identify changes of 35–40% in the assessed immune phenotypes. Even for these larger phenotypical differences, we would have an approximately 20% chance of missing the association in our study design, which underscores the importance of independent replicative studies to confirm published observations. Although we cannot exclude the possibility of smaller effects our data support that, if present, alterations in the composition of the peripheral B cell compartment in T1D patients are subtle and, as a consequence, of limited utility as a biomarker for the clinical stratification of T1D patients.

Taken together, our data suggest that there is no obvious defect in the B cell compartment in the peripheral blood of T1D patients after disease diagnosis. However, we cannot rule out a putative role of these subsets in different stages of disease, namely in the prediabetic stages – most notably at the time of seroconversion – or at the time of disease diagnosis. In addition, our findings are limited to the delineation of B cell subsets in peripheral blood, as it is the most accessible source of lymphocytes in humans and, therefore, we cannot exclude the possibility that local alterations of specific B cell populations in lymphoid tissues can be implicated in the pathogenesis of the disease. Consistent with this hypothesis, Rituximab, a B cell-depleting therapeutic antibody, has shown some clinical efficacy in T1D in patients treated within 11 weeks of diagnosis [43]. Clinical efficacy was restricted to the first 3 months following the initiation of the treatment course, with a similar decrease in the C-peptide levels being observed between the treated and the placebo groups thereafter, although the potential therapeutic effect of repeated treatment courses remains to be established. These data suggest that a pathogenic role of B cells in T1D may be limited to the earlier stages of the disease and locally in the islets of Langerhans and in the draining pancreatic lymph nodes. Moreover, T1D has been shown to develop in a case-study of a patient with severe B cell deficiency [44], suggesting that B cells are not responsible for disease pathogenesis in every T1D patient. Other human autoimmune diseases, including systemic lupus erythematosus, rheumatoid arthritis, Sjögren's syndrome and autoimmune thrombocytopenia [18,19,23], have been linked with a CD19^+^CD27^−^CD24^hi^CD38^hi^ B cell deficiency. Consistent with this suspected pathogenic role of this subset in these conditions, diagnosis of these diseases occurs typically later in life, at a stage where CD19^+^CD27^−^CD24^hi^CD38^hi^ cell numbers have already declined sharply. In contrast, T1D is most prevalent among children and young adults, and the observed decline of CD19^+^CD27^−^CD24^hi^CD38^hi^ B cells with age does not mirror an increased risk of T1D, further arguing against a major role of CD19^+^CD27^−^CD24^hi^CD38^hi^ B cells in the pathogenesis of the disease.

In contrast to the frequency of circulating B cells, our data suggest that genetically regulated alterations in the production of IL-10 could have a role in the pathogenesis of the disease. Consistent with this hypothesis, T cell-mediated antigen-specific responses in T1D patients have been shown to be polarized to an inflammatory IFN-γ response compared to a regulatory, IL-10-driven response in healthy donors [31]. Furthermore, a subset of T1D patients with later disease onset were found to maintain a higher level of IL-10 secreting CD4^+^ T cells compared to patients with more rapid disease onset and progression [45]. These data support that the balance between proinflammatory, IFN-γ-mediated and regulatory IL-10-mediated immune responses could be under the genetic control of disease-associated loci, including the *IL2–IL21* locus, which we have characterized in this study. To our knowledge, this is the first evidence showing that genetic regulation of a cytokine regulates the production of another cytokine located in a different genomic locus. Genetic studies have implicated altered IL-2 signalling in the aetiology of T1D [1], and both memory B cells and activated T cells, where we found evidence for genetic regulation of IL-10 production, express high levels of the high-affinity IL-2 receptor α-chain (IL-2RA). In addition, IL-2 signalling through the IL-2RA receptor has been shown previously to enhance IL-10-mediated activation of humoral immune responses [46], suggesting an interaction between IL-2 signalling and IL-10 production. Similarly, IL-21 has been shown to induce IL-10 mRNA and protein expression in both B [20] and T cells [47]. Further work will therefore be required to validate this association and, if confirmed, to characterize the functional mechanisms leading to impaired production of the anti-inflammatory cytokine IL-10 in individuals carrying the *IL2–IL21* T1D risk allele.

## Supporting information

**Fig. S1.** Age-dependent changes in the absolute number of B cell subsets. Scatter-plots depict the linear regression of the effect of age (represented on the *x*-axis) on the absolute number of total CD19^+^ (*n* = 55) (a), naive CD19^+^ (*n* = 55) (b), memory CD19^+^ (*n* = 55) (c), transitional CD19^+^CD27^−^CD24^hi^CD38^hi^ (*n* = 55) (d), CD5^+^ (*n* = 44) (e) and CD19^+^CD27^+^immunoglobulin (Ig)D^+^IgM^+^ marginal zone (*n* = 55) (f). B cells (represented on the *y*-axis) in 55 of the 91 donors included in this study for whom we had access to full blood counts. ρ = correlation coefficient.

**Fig. S2.** Rate of age-dependent changes in the frequency of B cell subsets does not differ between type 1 diabetes (T1D) patients and healthy controls. Scatter- plots depict the age-dependent changes in the frequency of the six assessed B cell subsets in T1D patients (depicted in red) and healthy controls (depicted in light green). The linear regression line is represented for T1D patients and controls. *P*-values were calculated by testing the interaction between age and disease status.

**Table S1.** Antibodies and immunostaining panels used for flow cytometry. **Table S2.** Reproducibility of the assessed B-cell subsets. **Table S3.** Association of the B-cell phenotypes with duration of disease.
