# Supplementary figures and images for: Multi-parametric flow cytometric and genetic investigation of the peripheral B cell compartment in human type 1 diabetes
Source: Clin Exp Immunol. 2014 Jul 24;177(3):571–85. doi: 10.1111/cei.12362 (PMC4137841; doi:10.1111/cei.12362)

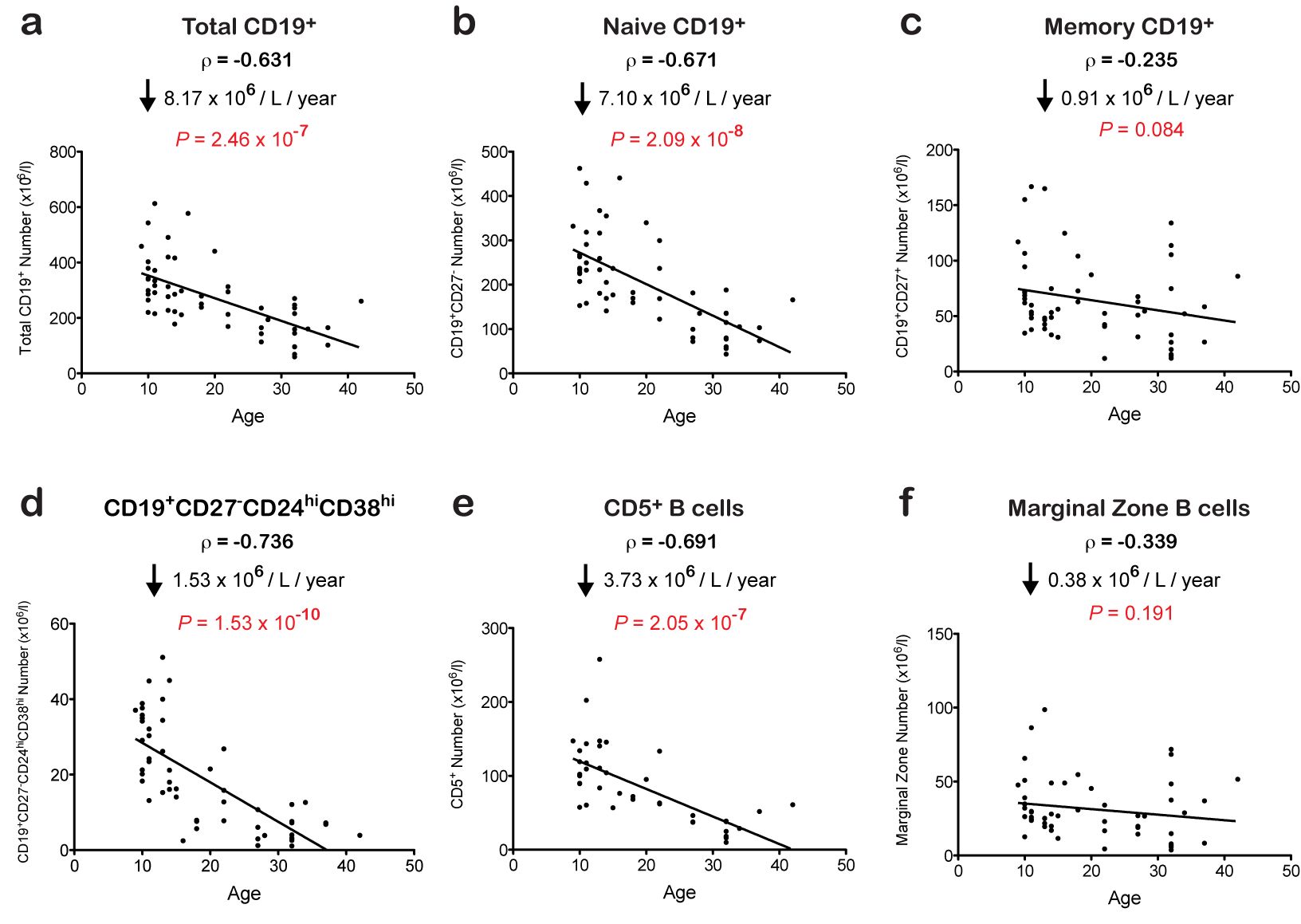

Supplement: Supplementary file 1 — Fig. S1. Age-dependent changes in the absolute number of B cell subsets. Scatter-plots depict the linear regression of the effect of age (represented on the x-axis) on the absolute number of total CD19+ (n = 55) (a), naive CD19+ (n = 55) (b), memory CD19+ (n = 55) (c), transitional CD19+CD27−CD24hiCD38hi (n = 55) (d), CD5+ (n = 44) (e) and CD19+CD27+immunoglobulin (Ig)D+IgM+ marginal zone (n = 55) (f). B cells (represented on the y-axis) in 55 of the 91 donors included in this study for whom we had access to full blood counts. ρ = correlation coefficient. [file cei0177-0571-SD1.jpg]

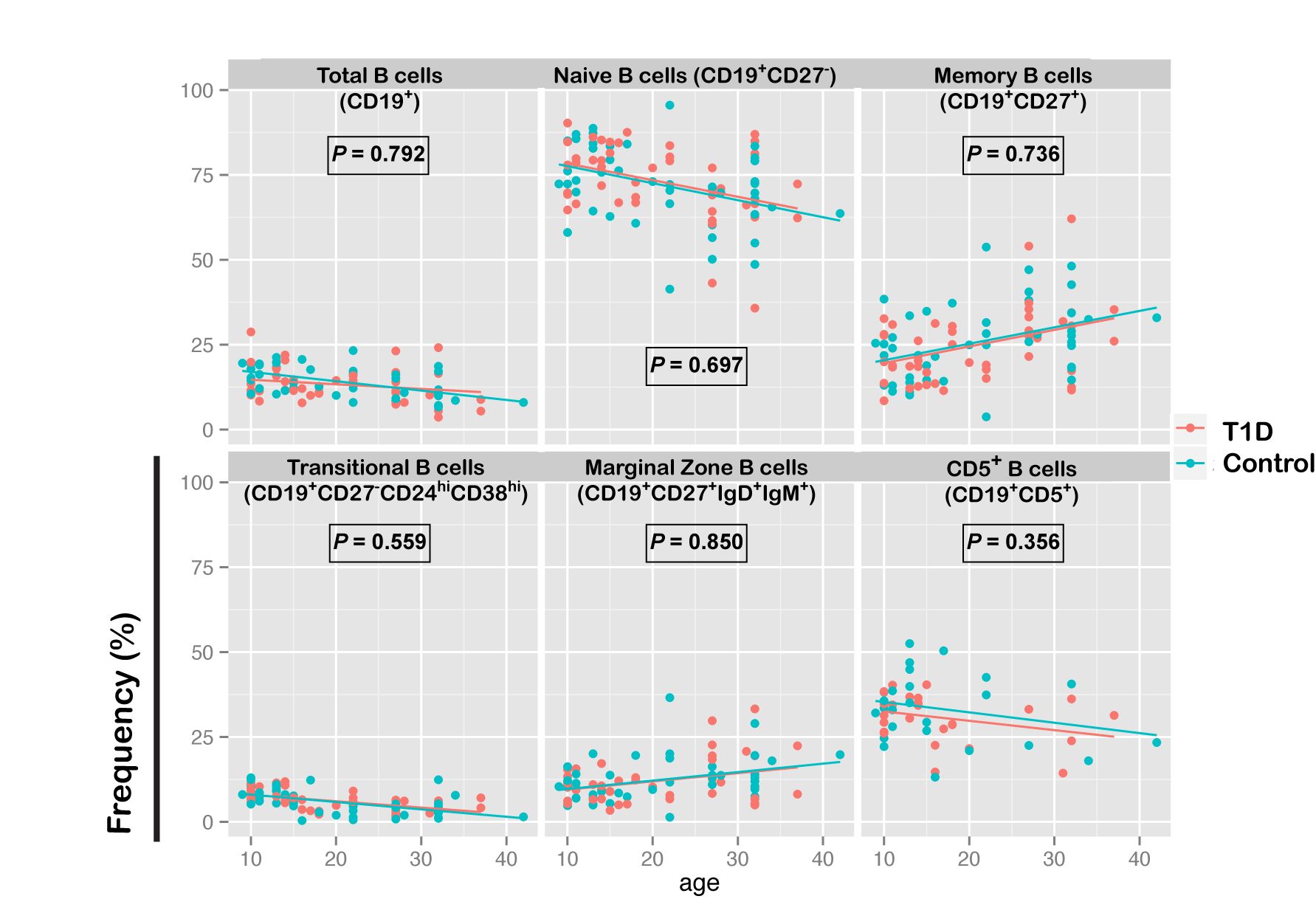

Supplement: Supplementary file 2 — Fig. S2. Rate of age-dependent changes in the frequency of B cell subsets does not differ between type 1 diabetes (T1D) patients and healthy controls. Scatter- plots depict the age-dependent changes in the frequency of the six assessed B cell subsets in T1D patients (depicted in red) and healthy controls (depicted in light green). The linear regression line is represented for T1D patients and controls. P-values were calculated by testing the interaction between age and disease status. [file cei0177-0571-SD2.jpg]
