# Supplementary material for: Multi-parametric flow cytometric and genetic investigation of the peripheral B cell compartment in human type 1 diabetes
Source: Clin Exp Immunol. 2014 Jul 24;177(3):571–85. doi: 10.1111/cei.12362 (PMC4137841; doi:10.1111/cei.12362)
Supplement: Supplementary file 3 [file cei0177-0571-SD3.pdf]

Table S1. Antibodies and immunostaining panels used for flow cytometry.

Immunostaining Panel	Antibody	Fluorochrome	Clone	Manufacturer
**Surface Panel 1**	CD19	AF700	HIB19	BioLegend
	CD27	BV421	O323	BioLegend
	CD38	PE-Cy7	HIT2	BioLegend
	CD24	FITC	ML5	BioLegend
	IgD	PerCP-Cy5.5	IA6-2	BD Biosciences
	IgM	APC	MHM-88	BioLegend
	IL-21R	PE	2G1-K12	BioLegend
**Surface Panel 2**	CD19	AF700	HIB19	BioLegend
	CD27	BV421	O323	BioLegend
	CD38	PE-Cy7	HIT2	BioLegend
	CD24	FITC	ML5	BioLegend
	CD5	PE	UCHT2	BioLegend
	CD25*	APC	M-A251+2A3	BD Biosciences
**Intracellular Panel 1**	CD19	AF700	HIB19	BioLegend
	CD27	BV421	O323	BioLegend
	CD38	PE-Cy7	HIT2	BioLegend
	CD24	FITC	ML5	BioLegend
	IgM	APC	MHM-88	BioLegend
	IL-10 (IC)	PE	JES3-19F1	BioLegend
	Viability Dye	eFluor780	-	eBioscience

Anti-human monoclonal antibodies used for the B-cell immunophenotyping. *2 clones of anti-CD25 that bind to different epitopes were used simultaneously to enhance CD25 staining. IC, Isotype control.

Table S2. Reproducibility of the assessed B-cell subsets

Phenotype	Delineation markers	N	ρ
Total $CD19^+$ B cells	$CD19^+$	18	0.778
Naïve B cells	$CD19^+CD27^-$	18	0.995
Memory B cells	$CD19^+CD27^+$	18	0.999
Anergic B cells	$CD19^+CD27^-IgD^+IgM^-$	18	0.359
Transitional B cells	$CD19^+CD27^-CD24^{hi}CD38^{hi}$	18	0.963
Marginal zone B cells	$CD19^+CD27^+IgD^+IgM^+$	18	0.925
$CD5^+$ B cells	$CD19^+CD5^+$	N/A	N/A

Reproducibility of the assessed B-cell phenotypes was determined by comparing repeated measurements from 18 donors performed between 1-2 months after the original measurement. ρ, correlation coefficient; N/A, not available.

Table S3. Association of the B-cell phenotypes with duration of disease.

Phenotype	N	*P value*	β (95% CI)
B cell subsets			
Total $CD19^+$ B cells	43	0.812	0.002 (-0.019 / 0.023)
Naïve B cells ($CD27^-$)	43	0.757	0.008 (-0.066 / 0.051)
Memory B cells ($CD27^+$)	43	0.856	0.004 (-0.051 / 0.060)
Transitional B cells ($CD27^-CD24^{hi}CD38^{hi}$)	43	0.358	0.007 (-0.025 / 0.011)
Marginal zone B cells ($CD27^+IgD^+IgM^+$)	43	0.360	0.014 (-0.022 / 0.050)
$CD5^+$ B cells	28	0.087	0.061 (-0.025 / 0.147)
IL-10 production[1]			
$IL\text{-}10^+$ Total B cells	33	0.388	-0.0017 (-0.0063 / 0.0030)
$IL\text{-}10^+$ Naïve B cells	33	0.325	-0.0016 (-0.0054 / 0.0023)
$IL\text{-}10^+$ Memory B cells	33	0.389	-0.0021 (-0.0079 / 0.0037)

Effect of duration of disease was calculated using a linear regression model including age and batch as covariates. β = beta coefficient and 95% confidence interval (CI) of the linear regression model. [1]Statistical tests were performed on log-transformed data because IL-10 phenotypes showed a strong right skew.
